# Intake of total and selected carotenoids and colorectal cancer risk: An Italian case-control study

**DOI:** 10.1038/s41430-025-01661-7

**Published:** 2025-09-17

**Authors:** Arianna Natale, Angela D’Angelo, Ettore Bidoli, Federica Toffolutti, Attilio Giacosa, Livia S. A. Augustin, Eva Negri, Francesca Bravi, Carlo La Vecchia, Marta Rossi

**Affiliations:** 1 Department of Clinical Sciences and Community Health – Dipartimento di Eccellenza 2023-2027, University of Milan, Milan, Italy; 2 Cancer Epidemiology Unit, Centro di Riferimento Oncologico di Aviano (CRO) IRCCS, Aviano, Italy; 3 Unit of Digestive Trait Endoscopy, CDI (Centro Diagnostico Italiano), Milan, Italy; 4 Epidemiology and Biostatistics Unit, Istituto Nazionale Tumori, IRCCS Fondazione G. Pascale, Naples, Italy; 5 Department of Medical and Surgical Science, University of Bologna, Bologna, Italy

**Keywords:** Risk factors, Cancer prevention, Cancer epidemiology

## Abstract

**Background:**

Colorectal cancer (CRC) risk is influenced by diet. Carotenoids are naturally occurring pigments primarily found in fruits and vegetables. Their potential chemopreventive properties are due to antioxidant, antimutagenic, and antiproliferative characteristics.

**Objectives:**

We investigated dietary carotenoid intakes (α-carotene, β-carotene, β-cryptoxanthin, lycopene, lutein+zeaxanthin, and total carotenoids) in relation to CRC risk.

**Methods:**

We used data from a case-control study on CRC conducted in Italy, which included 1953 histologically confirmed incident cases of CRC and 4154 controls. For each subject, carotenoid intake was estimated through a reproducible and valid food frequency questionnaire, using an Italian food composition database. Odds ratios (OR) and the corresponding 95% confidence intervals (95% CI) of CRC for the highest versus the lowest quintiles of carotenoid intakes were computed through multiple logistic regression models, including terms for total energy intake and other selected confounding factors.

**Results:**

The OR of CRC for the highest versus the lowest quintile was 0.72 (95% CI = 0.60–0.87) for α-carotene, 0.60 (95% CI = 0.49–0.73) for β-carotene, 0.83 (95% CI = 0.69–0.99) for β-cryptoxanthin, 0.64 (95% CI = 0.53–0.78) for lutein+zeaxanthin, and 0.59 (95% CI = 0.48–0.73) for total carotenoids, with significant trends across quintiles. No significant association was found for lycopene.

**Conclusions:**

Our findings indicate an inverse association between total and selected carotenoids and CRC risk.

## Introduction

Colorectal cancer (CRC) risk is influenced by several modifiable lifestyle factors, including alcohol, tobacco, overweight and obesity, physical activity, and several aspects of diet [1–3]. Diets high in fruit and vegetables and low in red and processed meat have been associated with lower CRC risk [1]. Identifying which dietary compounds play a key role against CRC risk could be relevant for developing preventive strategies.

Carotenoids are naturally occurring pigments primarily found in fruits and vegetables, known for their antioxidant properties [4]. The major dietary carotenoids are α-carotene, β-carotene, β-cryptoxanthin (precursors of vitamin A), lycopene, lutein and zeaxanthin [5]. The benefits of carotenoids are attributed to their radical-quenching, antimutagenic, and antiproliferative properties [6]. Their anticancer properties could be partially due to the provitamin A activity. All-trans retinoic acid, a metabolite of carotenoids, inhibit HOX genes expression, possibly reducing CRC carcinogenesis [7]. Carotenoids can also improve gap junctional intercellular communication and induce connexin43 against cancer progression and metastasis [8].

There is some evidence of an inverse association between intake of α-carotene, β-carotene, β-cryptoxanthin and CRC risk [9]. However, most epidemiological studies were focused on total carotenoids or mainly on lycopene and β-carotene, whereas information on other carotenoids—such as β-cryptoxanthin and lutein+zeaxanthin—is limited [9]. Moreover, high serum carotenoid levels were associated with reduced CRC risk and mortality [9, 10].

This study aims to systematically investigate dietary carotenoid intakes (α-carotene, β-carotene, β-cryptoxanthin, lycopene, lutein+zeaxanthin, and total carotenoids) in relation to CRC risk using data from a large Italian study.

## Methods

We analyzed data from a case-control study on CRC, conducted between 1992 and 1996 in the Italian areas of Milan, Genoa, Naples, and in the provinces of Forlì, Latina and Pordenone-Gorizia [11]. Participants were 1953 histologically confirmed incident cases of CRC (1225 with colon and 728 with rectal cancer), aged 19–74 (median age 62), admitted to major general and teaching hospitals. Controls were 4154 patients aged 20–74 (median age 58) with no previous history of cancer, admitted to the same hospitals as cases for acute conditions unrelated to neoplastic conditions, to known risk factors for CRC or to long-term diet alterations. Controls were admitted for traumas as sprains and fractures (23%), other orthopedic conditions (28%), acute surgical conditions (20%), eye disorders (19%) and miscellaneous other illnesses like dental, ear, nose and throat or skin conditions (10%). Less than 5% of the subjects contacted refused to participate in this study.

Centrally trained interviewers administered a structured questionnaire to collect data on sociodemographic characteristics, anthropometric measures, lifestyle factors and family history of cancer. The lifestyle section included questions on tobacco smoking (smoking status, duration, and average number of cigarettes, cigars or pipe per day); on alcohol consumption, (duration, frequency, and types of alcoholic beverages per week); and on occupational physical activity (mainly sitting or standing - low, average - moderate, heavy or strenuous - high/very high job activities). Information on the habitual diet before cancer diagnosis (for cases) or hospital admission (for controls) was collected using a food frequency questionnaire (FFQ), tested for validity [12] and reproducibility [13, 14]. The FFQ included questions about the average weekly consumption of 78 food or recipes items plus 5 questions on the consumption of alcoholic drinks. Intakes greater or equal to once per month and lower than once per week were coded as 0.5 times per week.

For each subject, the intakes of selected carotenoids (i.e., α-carotene, β-carotene, β-cryptoxanthin, lycopene, lutein+zeaxanthin, and total carotenoids) were estimated using an Italian food composition database [15]. Similarly, dietary fiber and total energy intakes were assessed [15].

### Statistical analysis

The odds ratios (OR) of CRC and the corresponding 95% confidence intervals (CI) were estimated for the highest versus the lowest quintiles of carotenoid intake (computed among controls) using multiple logistic regression models. The models included terms for study center, year of interview (continuously), sex, age (quinquennia), years of education (<7, 7-11, ≥12), body mass index (BMI, <25, 25- < 30, ≥30 kg/m^2^), tobacco smoking status (never, current, former smoker, categorically), alcohol consumption (0, >0- < 2, 2- < 4, 4- < 8, ≥8 drinks per week), occupational physical activity (low, moderate, high/very high), family history of CRC and total energy intake (quintiles among controls, categorically). We further considered a model including terms for dietary fiber intake (continuously). P-values for trend across quintiles were obtained through the Wald ${{\rm{\chi }}}_{1}^{2}$ test.

The OR of CRC for an increment equal to one standard deviation (SD, computed on controls), were also estimated. Additional analyses for colon and rectal cancers separately were performed. Moreover, we conducted stratified analyses by sex, age (<60, ≥60), BMI (<25, ≥25 kg/m^2^), tobacco smoking status (never, current, former smokers) and alcohol consumption (0- <2, 2- < 7, ≥7 drinks per week). Likelihood ratio test was used to assess heterogeneity across strata.

All the analyses were performed with SAS software version 9.4 (SAS Institute, Inc.).

## Results

Supplementary Table 1 reports the distribution of 1953 CRC cases and 4154 controls by major sociodemographic and lifestyle factors. Cases were more frequently males, older, and more likely to report a higher education level and a family history of CRC compared to controls.

Table 1 shows the mean ( ± SD) of carotenoid intakes and the ORs with the corresponding 95% CIs of CRC for the higher versus the lowest quintiles. The mean intake of carotenoids among controls was 765 (±839) μg/day for α-carotene, 4830 (±2611) μg/day for β-carotene, 333 (±283) μg/day for β-cryptoxanthin, 7153 (±3461) μg/day for lycopene, 4821 (±2538) μg/day for lutein+zeaxanthin, and 17902 (±6961) μg/day for total carotenoids. There was an inverse association of α-carotene, β-carotene, β-cryptoxanthin, lutein+zeaxanthin, and total carotenoid intake with CRC risk, with significant trend across quintiles. No association was observed for lycopene. For α-carotene, the OR for the highest versus the lowest quintile of intake was 0.72 (95% CI = 0.60–0.87), and the continuous OR for an increment of a SD was 0.86 (95% CI = 0.79–0.92). For β-carotene, the corresponding ORs were 0.60 (95% CI = 0.49–0.73) and 0.80 (95% CI = 0.74–0.86); for β-cryptoxanthin, 0.83 (95% CI = 0.69–0.99) and 0.96 (95% CI = 0.90–1.02); for lutein+zeaxanthin, 0.64 (95% CI = 0.53–0.78) and 0.86 (95% CI = 0.81–0.92); for total carotenoids, 0.59 (95% CI = 0.48–0.73) and 0.86 (95% CI = 0.80–0.92). Supplementary Table 2 presents ORs of CRC with additional adjustment for dietary fiber. Findings were generally unchanged, although the OR for the highest versus the lowest quintile of β-cryptoxanthin was 0.87 (95% CI = 0.72–1.05).

**Table 1:** Odds ratios and the corresponding 95% confidence intervals of colorectal cancer according to quintiles of carotenoid intakes.

			*OR (95% CI)*^*a*^*, Quintiles*											
	Mean (SD)^b^		I	II		III		IV		V		*p* for trend	Continuous OR ^c^	
**α-carotene**														
	765.41	(839.39)												
Upper cutpoints (μg/day)			194.97	426.85		707.27		1160.32		-				
Cases, *n* (%)			469 (24.0)	410 (21.0)		391 (20.0)		355 (18.2)		328 (16.8)				
			1	0.93 (0.78-1.11)		0.91 (0.76-1.08)		0.77 (0.64-0.92)		0.72 (0.60-0.87)		*<.0001*	0.86	(0.79-0.92)
**β-carotene**														
	4830.33	(2611.48)												
Upper cutpoints (μg/day)			2903.22	3919.06		4909.77		6318.42		-				
Cases, *n* (%)			488 (25.0)	434 (22.2)		373 (19.1)		330 (16.9)		328 (16.8)				
			1	0.90 (0.76-1.07)		0.73 (0.61-0.88)		0.65 (0.54-0.79)		0.60 (0.49-0.73)		*<.0001*	0.80	(0.74-0.86)
**β-cryptoxanthin**														
	332.74	(283.09)												
Upper cutpoints (μg/day)			111.86	245.31		306.60		541.71		-				
Cases, *n* (%)			440 (22.5)	381 (19.5)		426 (21.8)		333 (17.1)		373 (19.1)				
			1	0.81 (0.68-0.97)		0.80 (0.67-0.95)		0.70 (0.58-0.84)		0.83 (0.69-0.99)		*0.0095*	0.96	(0.90-1.02)
**Lycopene**														
	7152.73	(3461.01)												
Upper cutpoints (μg/day)			4295.99	5982.58		7584.40		9755.38		-				
Cases, *n* (%)			361 (18.5)	385 (19.7)		420 (21.5)		375 (19.2)		412 (21.1)				
			1	1.04 (0.86-1.25)		1.08 (0.90-1.30)		0.99 (0.81-1.20)		1.10 (0.90-1.35)		*0.5251*	1.03	(0.96-1.10)
**Lutein+zeaxanthin**														
	4820.95	(2537.57)												
Upper cutpoints (μg/day)			2745.74	3968.46		5048.18		6439.95		-				
Cases, *n* (%)			467 (23.9)	450 (23.0)		374 (19.2)		337 (17.3)		325 (16.6)				
			1	0.95 (0.80–1.13)		0.77 (0.64-0.92)		0.73 (0.61-0.88)		0.64 (0.53-0.78)		*<.0001*	0.86	(0.81-0.92)
**Total carotenoids**														
	17902.16	(6961.21)												
Upper cutpoints (μg/day)			12389.39	15623.42		18707.09		22846.58		-				
Cases, *n* (%)			453 (23.2)	396 (20.3)		405 (20.7)		382 (19.6)		317 (16.2)				
			1	0.81 (0.68–0.98)		0.82 (0.68-0.99)		0.79 (0.65-0.96)		0.59 (0.48-0.73)		*<.0001*	0.86	(0.80-0.92)

*SD* standard deviation, *OR* odds ratio, *CI* confidence interval.

^a^ Estimated using a logistic regression model adjusted for study center, year of interview, sex, age, years of education, body mass index, tobacco smoking status, alcohol consumption, occupational physical activity, family history of colorectal cancer and total energy intake.

^b^ Computed among controls.

^c^ Estimated for an increment of intake equal to one SD (computed among controls).

Table 2 reports the ORs of colon and rectal cancer for the highest versus the lowest quintile of carotenoid intakes. For α-carotene, the ORs of colon and rectal cancer were respectively 0.72 (95% CI = 0.57–0.89) and 0.73 (95% CI = 0.55–0.95); for β-carotene, the corresponding ORs were 0.65 (95% CI = 0.52–0.82) and 0.52 (95% CI = 0.39–0.69); for β-cryptoxanthin, 0.92 (95% CI = 0.74–1.13) and 0.71 (95% CI = 0.54–0.92); for lycopene, 1.05 (95% CI = 0.83–1.34) and 1.21 (95% CI = 0.90–1.62); for lutein+zeaxanthin, 0.63 (95% CI = 0.50–0.79) and 0.66 (95% CI = 0.50–0.86); for total carotenoids, 0.62 (95% CI = 0.48–0.79) and 0.56 (95% CI = 0.41–0.75).

**Table 2:** Odds ratios and corresponding 95% confidence interval of colon and rectal cancer for the highest versus the lowest quintile of carotenoid intakes.

	*OR*^*a*^ *for V vs I quintile (95% CI)*	
	Colon	Rectum
**α-carotene**	0.72 (0.57–0.89)	0.73 (0.55–0.95)
**β-carotene**	0.65 (0.52–0.82)	0.52 (0.39–0.69)
**β-cryptoxanthin**	0.92 (0.74–1.13)	0.71 (0.54–0.92)
**Lycopene**	1.05 (0.83–1.34)	1.21 (0.90–1.62)
**Lutein+zeaxanthin**	0.63 (0.50–0.79)	0.66 (0.50–0.86)
**Total carotenoids**	0.62 (0.48–0.79)	0.56 (0.41–0.75)

*OR* odds ratio, *CI* confidence interval.

^a^ Estimated using a logistic regression model adjusted for study center, year of interview, sex, age, years of education, body mass index, tobacco smoking status, alcohol consumption, occupational physical activity, family history of colorectal cancer and total energy intake.

**Fig. 1:**
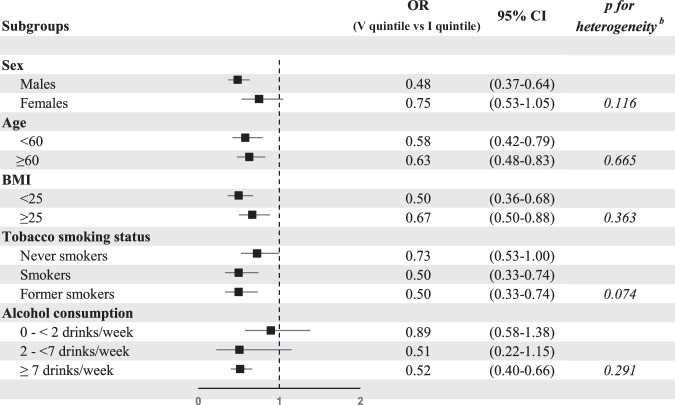
Odds ratios ᵃ and the corresponding 95% confidence intervals of colorectal cancer for the highest versus the lowest quintile of total carotenoid intakes in strata of selected factors. OR odds ratio, CI confidence interval, BMI body mass index. ^a^ Estimated using a logistic regression model adjusted for study center, year of interview, sex, age, years of education, BMI, tobacco smoking status, alcohol consumption, occupational physical activity, family history of colorectal cancer and total energy intake. ^b^ *p* of test for heterogeneity.

Figure 1 gives the OR of CRC for the highest versus the lowest quintile of total carotenoid intakes in strata of sex, age, BMI, smoking status, and alcohol consumption. The OR was 0.48 (95% CI = 0.37–0.64) among males and 0.75 (95% CI = 0.53–1.05) among females; 0.58 (95% CI = 0.42–0.79) among <60 years and 0.63 (95% CI = 0.48–0.83) among ≥60 years old; 0.50 (95% CI = 0.36-0.68) among BMI < 25 kg/m^2^ and 0.67 (95% CI = 0.50–0.88) among ≥25 kg/m^2^; 0.73 (95% CI = 0.53–1.00) among never smokers, 0.50 (95% CI = 0.33–0.74) among smokers and 0.50 (95% CI = 0.33–0.74) among former smokers; 0.89 (95% CI = 0.58–1.38) among individuals who drank 0- < 2 drinks/week; 0.51 (95% CI = 0.22–1.15) among 2- < 7 drinks/week and 0.52 (95% CI = 0.40–0.66) among ≥7 drinks/week. No heterogeneity was found between strata considered.

## Discussion

This study found an inverse association between intakes of α-carotene, β-carotene, β-cryptoxanthin, lutein+zeaxanthin, and total carotenoids and CRC risk, with a linear trend for most of them. The inverse associations were also observed separately for colon and rectal cancer. When we examined population strata by sex, age, BMI, smoking status, and alcohol consumption, no material differences were observed.

Carotenoids mostly exhibit antitumor effects due to their antioxidant properties, as well as to their ability to modulate cell signaling, inhibit cell cycle progression, modulate immune responses, and promote apoptosis [8]. Supplementation with α-carotene, lycopene and lutein in rats resulted in a protective effect against the development of aberrant crypt foci [16]. Studies in human colon cancer (CC) cells and in mouse CC models [17] showed that β-carotene inhibited cell proliferation through the suppression of M2 macrophage markers expression. β-carotene can also upregulate BCMO1 and inhibit MMP7 and MMP28 expression, reducing human CC cell invasiveness and migration ability [18]. In rats, citrus juices and pulp rich in β-cryptoxanthin inhibited CC progression, decreased the expression of the mRNAs of inflammatory factors as COX-2, TNF-α, IL6 and IL1-β and promoted the expression of mRNA of Nrf2, a cytoprotective transcription factor [8]. Lycopene reduced Akt activation and non-phosphorylated β-catenin expression in human CRC cells, while increased phosphorylated β-catenin levels, which is associated with reduced cyclin D1 expression and to reduced CRC growth and proliferation [19]. Moreover, in humans mucosal β-carotene, β-cryptoxanthin, lycopene and zeaxanthin were lower in adenoma tissue compared to non-involved mucosa [20]. Serum carotenoids were found to be lower in adenoma as compared to healthy subjects [21].

A meta-analysis on the relationship between dietary carotenoids and CRC risk, including approximately 8900 North American, 5300 European, and 2450 Asian cases, reported an inverse association for α-carotene (OR for high versus low intake: 0.87, 95% CI = 0.72–1.03), β-carotene (OR: 0.89, 95% CI = 0.78–1.03) and β-cryptoxanthin (OR: 0.70, 95% CI = 0.48–1.01), and a null association for lycopene, lutein/zeaxanthin and total carotenoids [9]. Our data confirm the inverse association between α-carotene, β-carotene, β-cryptoxanthin intakes and CRC, and showed a somewhat stronger association than that reported in the meta-analysis [9]. Regarding lycopene intake, our data showed no association with CRC, in line with the literature [9, 22]. Blood concentration of lycopene also does not seem related to CRC risk, although the evidence is limited [9].

Higher lutein+zeaxanthin intake was associated with a lower CRC risk in our data. In the meta-analysis [9], most studies separately analyzed lutein intake and zeaxanthin intake, instead of lutein+zeaxanthin intake [23–26], without reaching the amount of lutein and zeaxanthin intakes of our study, which showed an inverse association from 5048 μg/day (III quintile). This suggests that the inverse association of lutein and zeaxanthin with CRC risk may not be evident for lower intakes.

For total carotenoids, we found an inverse association with CRC risk. There was a significant trend across quintiles, and we observed a risk reduction of ~20% with intakes higher than 15,623 μg/day (II quintile) and of ~40% with intakes higher than 22,847 μg/day (V quintile). A comparison with previous studies is difficult because estimates of total carotenoids intake were not consistent [25, 27–31]: some studies considered only selected carotenoids, β-carotene alone, or β-carotene equivalents [9]. Moreover, studies conducted in regions where carotenoid intake is lower, such as the U.S. and Asian countries [32, 33], did not reach our levels of total carotenoids intake for which we observed a reduction in CRC risk.

In our population, major carotenoid sources were carrots, citrus fruits, leaf vegetables, tomatoes, and peas. Our findings indicate therefore that a diet rich in a variety of fruits and vegetables provides an adequate intake of all carotenoids. Dietary fiber, which is well represented in carotenoids rich foods, has been also associated with a reduced CRC risk [3] and can act as a confounder. However, in our study, further adjustment for fiber intake did not materially modify the association (Supplementary Table 2). Differences in dietary carotenoids sources in other populations may explain the heterogeneity between studies. For example, industrially processed foods are relevant sources of carotenoids in countries such as the UK and Germany [34] and are considered risk factors for CRC [35, 36].

To reduce selection bias, cases and controls were enrolled in similar catchment areas and interview settings, and controls were selected among patients admitted to hospitals for acute, non-neoplastic conditions unrelated to diet. We took care to exclude individuals admitted for a diagnosis associated with known CRC risk factors or long-term dietary changes. Main strengths of this study were the participation rate, over 95%, and the large sample size. To record dietary habits, we used a reproducible [13] FFQ that has been validated [12] for the assessment of several nutrients, including β-carotene. Data were collected in the 1990s and concerns could be raised on the generalizability of dietary and lifestyle information. However, fruit and vegetable consumption did not substantially change across the 1990s and 2000s in Italy [37]. In addition, we were able to control for major confounders such as BMI, tobacco smoking, alcohol consumption, and total energy intake.

In conclusion, our findings indicate an inverse association between most carotenoids and the risk of CRC, supporting a variety of fruits and vegetables in dietary recommendations for CRC prevention.

## Supplementary information

Supplementary Table 1

Supplementary Table 2

## Data Availability

The data generated in the current study is available from the corresponding author upon reasonable request.
